# DGCPPISP: a PPI site prediction model based on dynamic graph convolutional network and two-stage transfer learning

**DOI:** 10.1186/s12859-024-05864-w

**Published:** 2024-07-31

**Authors:** Zijian Feng, Weihong Huang, Haohao Li, Hancan Zhu, Yanlei Kang, Zhong Li

**Affiliations:** 1 Zhejiang Province Key Laboratory of Smart Management and Application of Modern Agricultural Resources, School of Information Engineering, Huzhou University, Huzhou, 313000 Zhejiang China; 2 College of Science, Zhejiang Sci-Tech University, Hangzhou, 310018 Zhejiang China; 3 School of Mathematics, Physics and Information, Shaoxing University, Shaoxing, 312000 Zhejiang China

**Keywords:** PPI site prediction, Graph convolutional network, Transfer learning

## Abstract

**Background:**

Proteins play a pivotal role in the diverse array of biological processes, making the precise prediction of protein–protein interaction (PPI) sites critical to numerous disciplines including biology, medicine and pharmacy. While deep learning methods have progressively been implemented for the prediction of PPI sites within proteins, the task of enhancing their predictive performance remains an arduous challenge.

**Results:**

In this paper, we propose a novel PPI site prediction model (DGCPPISP) based on a dynamic graph convolutional neural network and a two-stage transfer learning strategy. Initially, we implement the transfer learning from dual perspectives, namely feature input and model training that serve to supply efficacious prior knowledge for our model. Subsequently, we construct a network designed for the second stage of training, which is built on the foundation of dynamic graph convolution.

**Conclusions:**

To evaluate its effectiveness, the performance of the DGCPPISP model is scrutinized using two benchmark datasets. The ensuing results demonstrate that DGCPPISP outshines competing methods in terms of performance. Specifically, DGCPPISP surpasses the second-best method, EGRET, by margins of 5.9%, 10.1%, and 13.3% for F1-measure, AUPRC, and MCC metrics respectively on Dset_186_72_PDB164. Similarly, on Dset_331, it eclipses the performance of the runner-up method, HN-PPISP, by 14.5%, 19.8%, and 29.9% respectively.

**Supplementary Information:**

The online version contains supplementary material available at 10.1186/s12859-024-05864-w.

## Introduction

Proteins, complex chains of amino acids, are fundamental in orchestrating most biological activities [1]. Intriguingly, proteins often do not operate in isolation but rather rely on protein–protein interactions (PPI) to effectuate cell functions [2]. PPI sites are the interfacial residues of a protein that interact with other protein molecules [3]. There are two main areas in PPI site prediction. One is pairwise interaction site prediction, which mainly predicts the interfacial residues of a pair of proteins [4]. The other, which is the topic of this paper, predicts the putative interaction sites when there is only an isolated protein without knowing the information of its partner protein or protein complex [5]. The latter prediction task is more formidable due to the paucity of information regarding its binding partner protein.

Currently, PPI site prediction methods are categorized into traditional biological methods and computational methods. The former, while reliable, are laborious and time-consuming, thus inadequate for addressing various research needs [6]. As a result, more expedient computational methods have achieved the rapid development. These can be segmented into three groups: (1) protein–protein docking and modeling; (2) structure-based methods and (3) sequence-based methods [7]. Although the first two groups often provide more comprehensive information than sequence-based methods, they depend on protein structure information, which is not available for all proteins. With the advent of high-throughput sequencing technology, acquiring protein sequences has become convenient, and sequence-based methods hold considerable promise in the field of PPI site prediction.

Machine learning techniques have been deployed in PPI site prediction. For instance, Guo et al. [8] formulated a feature space using sliding windows based on the influence of neighboring residues, and subsequently trained a support vector machine (SVM) model for PPI prediction. Wei et al. [9] devised a combination of SVM and random forest (RF) for PPI site classification. Zhang et al. [10] identified that cross-prediction of different protein ligands frequently occurs when predicting protein interaction residues. They then crafted a model based on Logistic Regression (LR) to mitigate this issue. These methods underscore the potential of computational approaches for PPI site prediction, but they fall short in deeply mining feature information due to inherent limitations of machine learning, thereby leading to limited generalization and sensitivity to imbalanced sample data.

Deep learning methods have also found applications in protein-related prediction. Their potent expressivity has proven increasingly pivotal in PPI site prediction in parallel with the exponential increase of biological data. For example, Zeng et al. [11] introduced DeepPPISP, a PPI site prediction model by combining local and global features with deep neural networks. Li et al. [12] proposed a DELPHI model integrating convolutional neural networks with recurrent neural networks [13] for PPI site prediction. Lu et al. [14] constructed a PPI site prediction model that uses an attention mechanism to capture the significance of residues at varying positions. Kang et al. [15] adopted a framework MLP-Mixer [16] to develop a two-stage multi-branching network, achieving superior PPI site prediction. All these methods rely on the sequence information of proteins, but they can not effectively capture the spatial structure features in proteins. Recently, data structures like graphs have been recognized as one of convenient and intuitive ways to represent residues in a protein, and their interactions [17]. Mahbub et al. [18] proposed an EGRET model, which introduces graph edge features based on graph attention networks (GAT) [19], while also incorporating encoding features of the pre-trained model ProtBERT through transfer learning [20]. Despite its efficacy, this transfer strategy was solely used in the feature extraction section. Transfer learning can improve the accuracy and generalization ability of the model by pre-training the model on a large dataset and applying it to a small dataset. The wide application of transfer learning in bioinformatics (gene expression prediction, cancer diagnosis, etc.) demonstrates its powerful ability in the case of insufficient data. Another issue is that graph neural networks pose limitations as they require prior construction of the protein's graph structure, with neighboring relationships based on the distance between residues. An improper threshold value for constructing edges would lead to erroneous or missing edges in the graph, thereby undermining the effectiveness of graph neural network. Dynamic graph convolution [21], a novel graph convolution pattern, can adaptively construct new graph structures based on the current feature information of the network, and can potentially address issues arising from fixed neighborhoods.

In PPI site prediction, the representation of protein sequences is also a critical factor, and feature selection directly influences the final outcome. Traditional features like the position-specific scoring matrix (PSSM) have proven effective in PPI site prediction. Though PSSM features for some known datasets have been provided by prior work (e.g., DeepPPISP [11], HN-PPISP [15]), PSSM computation for new protein sequences remains a lengthy process. Recently, Transformer and its variant models [22], trained on copious amounts of protein data using unsupervised methods, have been utilized as feature extractors of protein sequences. Their exceptional results and efficient extraction process underscore their substantial contribution to protein-related prediction. However, existing methods cannot make full use of pre-trained large model embeddings to migrate and characterize proteins, which to some extent reduces the generalization ability of the model and affects the PPI prediction accuracy.

To effectively address above issues we discuss, we introduce a two-stage transfer learning framework underpinned by dynamic graph convolutional neural network for PPI site prediction. The major contributions of this study include: (1) We encode the target sequence in the first stage of transfer learning using a protein pre-trained language model ESM-2 [23], coupled with four other sequence features as input to the training model. ESM-2 holds a wealth of latent information that can mitigate the inherent information deficiency in sequence-based methods. (2) We successfully find a protein-peptide binding residue dataset which is helpful for the PPI site prediction. To optimize the feature extraction module and enhance the final model's performance by supplying initial parameters, we pre-train the network using in the protein-peptide dataset and transfer the network parameters to the PPI site task for fine-tuning in the second stage of transfer learning. (3) When designing the neural network framework, we utilize the dynamic graph convolution module as the primary feature extraction technique, to address the limitation of traditional graph neural networks that cannot fully exploit the interaction information of neighboring nodes in the high-dimensional semantic space due to fixed adjacency relations.

## Datasets, materials and methods

### Datasets

This study leverages three distinct datasets, namely, the protein-peptide binding residue dataset (Dataset_trans) for protein transfer learning, and two PPI site datasets, Dset_186_72_PDB164 and Dset_331, for targeted task training and assessment. The criteria for defining a PPI site within these datasets is a surface residue (RSA > 5%) that sees a reduction in absolute solvent accessibility of more than 1 Å^2^ following the formation of a protein–protein complex [5].

1. Dataset_trans: A peptide, comprising a small aggregation of amino acids, shares chemical homogeneity with proteins. Our method necessitates the utilization of the protein-peptide binding residue dataset as proposed by SPRINT-Str [24] for our model's pre-training data. Herein, peptides are demarcated as chains with fewer than 30 amino acid residues. This dataset encompasses a total of 1,279 protein-peptide complexes, embodying 307,692 amino acid residues. Among these, 16,749 residues are categorized as binding residues, with remaining 290,943 residues classified as non-binding residues. From this dataset, we randomly select 10% of the complexes to form the test set, with the remaining complexes comprising the training set.
2. Dset_186_72_PDB164: To train and evaluate the model, we employ a dataset built by DeepPPISP [11], a standard dataset for PPI site prediction. This dataset amalgamates three benchmark datasets: Dset_186, Dset_72 [25], and PDBset_164 [26]. All three datasets are derived from the PDB database [27], maintaining a sequence homology under 25% and a resolution below 3.0 Å. For training and testing consistency and data augmentation, these three benchmark datasets are combined into a unified dataset containing 422 protein sequences. From this consolidated dataset, DeepPPISP randomly selects 70 protein sequences as a test set, with the remaining sequences allocated to training and validation sets. To ensure parity with various methods based on this dataset, we adopt the same partitioning strategy. Moreover, considering the homology impact of this dataset, we impose a sequence homology ceiling of 20% to curate a new version of Dset_186_72_PDB164 for PPI site prediction testing and comparison.
3. Dset_331: For broader evaluation of our model's performance, we also incorporate the Dset_331 dataset as proposed by HN-PPISP [15]. Derived from Dset_448 [10], this dataset encompasses 331 protein sequences, including 11,255 residues at PPI sites and 72,420 residues at non-PPI sites. Of all these proteins, 77 are selected for the test set, with the remainder designated for training and validation sets.

Detailed information about the PPI sites in these datasets is provided in Additional file 1: Table S1. The protein sequences in the PPI site datasets vary in length, as illustrated in Additional file 1: Table S2. To streamline training, we scrutinize the length distribution of protein sequences and determine that 96.7% of sequences in the datasets are shorter than 500 in length. Consequently, we standardize all sequences to a uniform length of 500.

### Feature extraction

Our model integrates five distinct features derived from protein sequences. These features are guided by their relevance and contribution for capturing biochemical properties of proteins, and their validities are corroborated in the ablation study of the subsequent experiment section. A detailed overview and selection process of these features are:

1. One-hot Encoding: This encoding is inherently sparse, memory-inefficient, high-dimensional, and there is no notion of similarity between sequence or structural elements. It can be denoted by a binary vector form composing of 0 s and 1 s, which is utilized to characterize the types of residues in a protein. Given the existence of 20 types of residues, each residue is individually encoded and subsequently merged into a feature vector of dimension *L* × 20, where *L* denotes the protein sequence's length.
2. Co-occurrence Similarity Encoding of Amino Acids: An amino acid co-occurrence is the occurrence of two amino acids in a protein segment, which can be used for reflecting the co-occurrence similarity of amino acids. This encoding can also be expressed by the vector form and acquired through pre-training a Skip-gram model grounded in protein sequences [28]. Each amino acid is thereby encoded as a word vector with a dimension of 5.
3. Similarity Encoding of Electrostaticity and Hydrophobicity [29]: The nature of protein–protein interactions is governed by their electrostatic and hydrophobic characteristics, which are embodied by the dipole and volume of amino acid side chains. As detailed in Additional file 1: Table S3, we categorize the 20 naturally occurring amino acids into 7 groups based on their side chain dipoles and volumes. For less common amino acids such as selenocysteine and pyrrolysine, they are assembled into category 8. Utilizing the one-hot encoding form, a final feature vector of dimension *L* × 8 is generated for similarity encoding of electrostaticity and hydrophobicity.
4. Position Encoding: It assigns each amino acid in the protein sequence with a vector representing its position, which has been established as an efficient descriptor in numerous protein-related applications [30]. In this way, the spatial information of the sequence is preserved, which is particularly important for the sequence structure and functional analysis. The coding scheme for position encoding is
  1 $$E\left( {pos,2i} \right) = \sin \frac{pos}{{b^{2i/d} }}$$
  2 $$PE\left( {pos,2i + 1} \right) = \cos \frac{pos}{{b^{2i/d} }}$$

where $pos$ indicates the position index of the current residue in the protein sequence (0 ≤ $pos$ ≤ $L -$ 1), $b$ and $d$ are two constants chosen as 1000 and 20 respectively in our model. Each residue is encoded as a 20-dimensional vector, and the exact position in the vector is indexed by the variables $2i$ and $2i + 1$ (0 ≤ *i* < 10). Finally, the position encoding of all residues in the sequence is combined into a feature vector with dimension *L* × 20.

(5) ESM-2 Encoding: Lin et al. [23] introduced a novel protein pre-trained language model based on an advanced Transformer architecture and leverages protein sequences from the UniRef database [31] for pre-training. The resulting pre-trained model maps raw sequences to biological feature representations without labels or prior knowledge. This facilitates a more comprehensive feature representation of protein sequence. In our model, we utilize an ESM-2 pre-trained model with 650 M parameters as a sequence encoder. Each amino acid residue is encoded as a 1280-dimensional vector, resulting in a protein sequence of length *L* with an encoding size of *L* × 1280.

### DGCPPISP prediction model

**Fig. 1:**
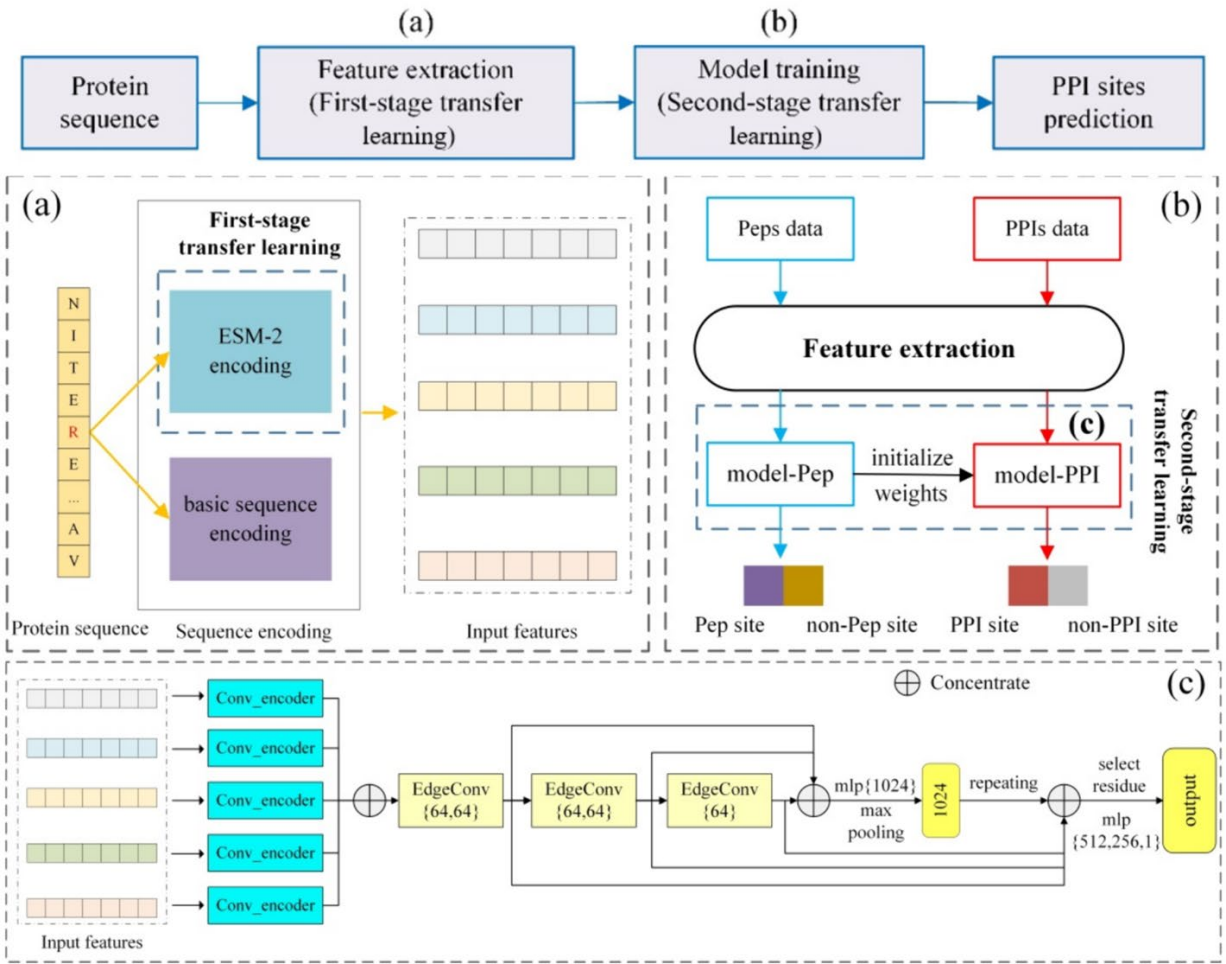
Schematic diagram of DGCPPISP. **a** Feature extraction. **b** Model training. **c** Network architecture for model-PPI. Sequence features in **a** include four features besides ESM-2 encoding. In **b**, Peps data represents protein sequences from the protein-peptide binding residue dataset, and PPIs data represents protein sequences from the PPI site prediction dataset. The model-Pep denotes the model pre-trained with Peps data and model-PPI denotes the final model for PPI site prediction after fine-tuning with PPIs data. **c** is the structure of model-PPI and model-Pep. Its input is five kinds of sequence encoding features. Conv_encoder uses a one-dimensional convolution to build a feature extraction and dimensionality reduction module. EdgeConv is the operation for dynamic graph convolution to update node features. The repeating operation means copying the feature vector to be consistent with the number of residues, “select residue” means to find the target residue from the input sequence

The main framework of our proposed PPI site prediction model, DGCPPISP, is exhibited in Fig. 1. It employs a two-stage transfer learning strategy for learning, with a dynamic graph convolution model for training implementation. For the two-stage transfer learning, they are manifested in the feature extraction module (Fig. 1a) and model training module (Fig. 1b). In the feature extraction, a pre-training model ESM-2 is utilized to encode protein sequences to furnish rich inherent information. For the model training, an additional protein-peptide binding residue dataset is employed to pre-train the model and optimize the initial model weights. Subsequently, we incorporate the EdgeConv module for dynamic graph convolution as the primary part of the second stage of transfer learning training (Fig. 1c). This dynamic graph operation enables the neural network to deeply analyze the interaction relationships between residues and ultimately assist in the prediction of PPI sites.

#### Two-stage transfer learning

We initially employ the first-stage transfer learning strategy in the feature extraction process (Fig. 1a) to fortify the initial feature representation derived from the primary structure of protein sequence. Specifically, we use the protein pre-trained model ESM-2 to encode protein sequences. As ESM-2 leverages a massive dataset to predict masked residues, its encoding allows the input sequence features to encapsulate more abundant information (such as secondary structure, protein interaction, etc.), thus enhancing the feature input of the sequence-based method.

During the model training, with the continuous deepening and expansion of data complexity, the learning of optimal parameters for the model becomes increasingly challenging. To effectively tackle this problem, we propose a second-stage transfer learning strategy (Fig. 1b) to discover suitable initial parameters for the PPI site prediction model, enabling the model to better adapt to the extraction of protein features. We first train the neural network on a protein-peptide binding site dataset (Peps data) akin to the PPI site prediction task. The model uses five protein sequence features as input, and the output is labeled 1 or 0 depending on whether the residues are identified as binding sites. After a certain number of iterations, we obtain the model-Pep. Then, we use the same network architecture and initialize model-PPI with the network weights of model-Pep, while lowering the learning rate and other parameters, allowing the new model to be retrained and fine-tuned on the PPI site task (PPIs data).

#### Dynamic graph convolutional module

When a traditional graph neural network addresses the problem of PPI site prediction, the graph structure of proteins is pre-constructed. Generally, the residue node features are used as the nodes of the graph, and the distance between residues and a given threshold are used to determine whether an adjacency relationship exists between two residues. The construction method is

3 $$G = \left( {\left\{ {v_{i} } \right\},\left\{ {e_{i,j}^{bond} |\left| {i - j} \right| = 1} \right\} \cup \left\{ {e_{i,j}^{contact} \left| {\left| {i - j} \right|} \right\rangle 1,\left| {\left| {C_{i} - C_{j} } \right|} \right| \le d_{max} } \right\}} \right)$$

where $e$ and $v$ are the edge and node of a graph, $i,j$ are the residue indices, *C* is the $C_{{\text{a}}}$ atomic coordinates in the residue (used to represent residue coordinates in our model), and $d_{max}$ is the threshold for constructing the edge. Although this representation has worked well for the protein prediction, this static adjacency relationship constructed by residue atom distances is not well suited for high-dimensional semantic spaces. Moreover, building a graph in this way often requires the protein structure information, which is not always easy to obtain for some proteins. A graph neural network that does not require pre-constructed graph structures and can dynamically construct neighborhoods based on the current feature representation becomes our motivation.

**Fig. 2:**
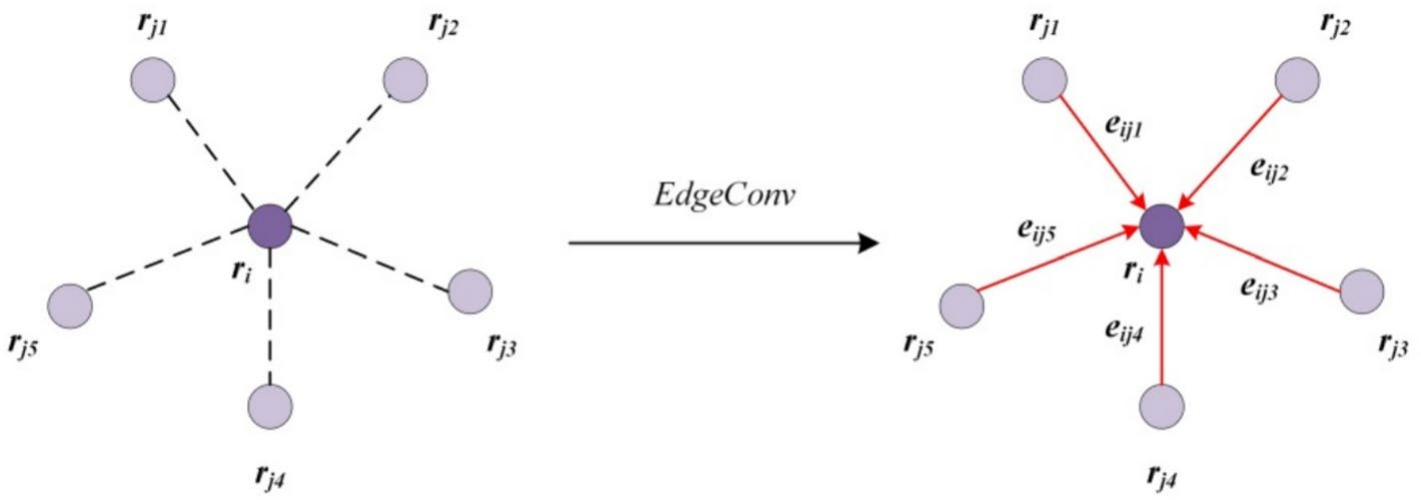
EdgeConv operation. The neighborhood range *k* = 5 in the figure, and node $r_{i}$ is updated by aggregating the features of neighbourhood node $r_{j}$ through the EdgeConv operation

The dynamism of the dynamic graph convolution is manifested in how it updates the features of residue nodes. The network does not aggregate features using a fixed adjacency matrix's node relationship but calculates the "feature distance" (Euclidean distance of the residue feature vector under the current network layer) between residues. It then constructs a local neighborhood centered on each node via the k-Nearest Neighbor (KNN) algorithm before each node update. As a result, residues located in the same local neighborhood are considered to be similar in the semantic space. The update of residue node features primarily relies on the EdgeConv operation as demonstrated in Fig. 2.

The operation can be defined by the following formula

4 $$r_{i}{\prime} = \mathop \rho \limits_{{j:\left( {i,j} \right) \in \varepsilon }} h_{\theta } \left( {r_{i} ,r_{j} } \right)$$

where $r_{i}{\prime}$ denotes the updated feature of residue $r_{i}$, $\rho$ represents the aggregation operation (specifically implemented as a MAX function in our model), $h_{\theta } \left( {r_{i} ,r_{j} } \right)$ denotes the function of edge feature $e_{ij}$ formed by the central node $r_{i}$ and its neighbor node $r_{j}$. $h_{\theta }$ is a non-linear function containing a learnable parameter $\theta$, and $\varepsilon$ denotes the set of edges formed by the central node and its neighbour nodes. In particular, there are many implementation forms of the edge function, and the specific choice $\overline{h}_{\theta }$ in our model is

5 $$h_{\theta } \left( {r_{i} ,r_{j} } \right) = \overline{h}_{\theta } \left( {r_{i} , r_{j} - r_{i} } \right)$$

where $r_{i}$ is the central node feature, which reflects the global feature information of the residue, and $r_{j} - r_{i}$ is the feature vector difference between the neighbouring nodes and the central node, which reflects the local details of the neighbourhood.

#### Neural network architecture for the second-stage transfer learning

Based on the EdgeConv operation, we construct a neural network module for PPI site prediction. This serves as the training component for the second-stage transfer learning (Fig. 1b), and its primary structure is depicted in Fig. 1c. Initially, each of five features is input to the Conv_encoder module of the network, comprising a one-dimensional convolution with a convolution kernel of length 3. The primary objective is to balance the dimensionality of five features. For instance, the initial dimension of ESM-2 encoding is L × 1280, which diverges significantly from the dimensionality of other features and could result in unbalanced feature impact issues. In our model, the feature output of Conv_encoder is set to a standard size of L × 32 and then amalgamated and provided to the EdgeConv layer.

The network's primary feature extraction module is a stack of three EdgeConv layers. Each EdgeConv layer generates a new protein dynamic graph structure before operation and transmits the updated graph to the next layer for use. Furthermore, inspired by the shortcut connection of ResNet [32], we adopt this structure and combine the feature vectors obtained by three EdgeConv modules to ensure the final classification has rich reference information without losing shallow features. It outputs a 1024-dimensional feature vector via convolution and pooling operations, which encompasses the key global information of the entire protein sequence. We concatenate the outputs of the first three layers of EdgeConv modules to represent residue features. As the network's ultimate goal is to predict the target residues in the protein sequence, the target residue feature vector is selected through the residue index saved in advance and merged with a 1024-dimensional protein global vector to form a batch size × 1216 vector. This vector is finally fed into the fully connected layer for classification.

### Implementation details

The pseudocode of the proposed DGCPPISP is illustrated in “Algorithm” (Supplementary Note part of the supplementary material). Our experiment leverages the Pytorch deep learning library for implementation. The Conv_encoder module uses a convolutional kernel length of 3, with a padding parameter set to 1. The activation function applied by all layers in the network is LeakyReLU, where the negative_slope parameter is 0.2, and the normalization layer is governed by Batch Normalization. Dropout is set to 0.5 in the fully connected layer. The neighborhood size (*k*) of each node in the EdgeConv operation is a hyperparameter that bears an impact on the model's performance. In our model, *k* is configured to 10. The multilayer perceptron (MLP) output dimension involved in the EdgeConv operation is 64, and the convolution kernel size is set to 1.

The optimization settings for the proposed model are as follows: The optimization algorithm is Adam [33], the learning rate (*lr*) is 0.01 in the initial stage of transfer learning, the *lr* for PPI site prediction is 0.001, and the batch size for network training is 32, the number of hidden layers is 3. We dynamically tune the learning rate by combining the grid search and StepLR methods with a range of [0, *lr*], a change step of 12, and a coefficient of variation (gamma) of 0.1. The training process adopts the F-measure [11] as the reference index, and the training is terminated when the F-measure has not improved for five consecutive epochs. The loss function used in our model is cross-entropy loss, and it is implemented as

6 $$loss = - \frac{1}{n}\sum \left[ {y\log \left( {y_{pred} } \right) + \left( {1 - y} \right)\log \left( {1 - y_{pred} } \right)} \right]$$

where $n$ is the number of all training data, $y$ is the true label and $y_{pred}$ is the predicted label.

## Experimental result and discussion

### Evaluation metrics

To evaluate the performance of DGCPPISP, seven common evaluation metrics are used in this paper: accuracy, precision, recall, F1-measure, area under the receiver operating characteristic curve (AUROC), area under the precision-recall curve (AUPRC) and Matthews correlation coefficient (MCC) [11]. The formulas of these metrics are

7 $${\text{A}}ccuracy = \frac{TP + TN}{{TP + TN + FP + FN}}$$

8 $$Precision = \frac{TP}{{TP + FP}}$$

9 $$Recall = \frac{TP}{{TP + FN}}$$

10 $$F - measure = \frac{2 \times Precision \times Recall}{{Precision + Recall}}$$

11 $$MCC = \frac{TP \times TN - FP \times FN}{{\sqrt {\left( {TP + FP} \right)\left( {TP + FN} \right)\left( {TN + FP} \right)\left( {TN + FN} \right)} }}$$

where $TP$ denotes correctly predicted PPI sites, $TN$ means correctly predicted non-PPI sites, $FP$ represents incorrectly predicted non-PPI sites and $FN$ is incorrectly predicted PPI sites. AUROC and AUPRC are metrics for the overall performance of the prediction model. Note that our model is implemented in the unbalanced datasets, we more focus on F1-measure, MCC, AUROC and AUPRC indices as the main evaluation besides accuracy, precision and recall metrics.

### Performance comparison and analysis with different methods

#### Comparison with competitive methods

In order to assess the efficacy of diverse prediction approaches, we carry out a series of experiments on PPI site datasets, Dset_186_72_PDB164 and Dset_331, by contrasting DGCPPISP with an array of current state-of-the-art methods. This comparison involves a total of 10 methods, six of which are structure-based (StackingPPINet [4], SPPIDER [34], DeepPPISP [11], Attention-CNN [14], HN-PPISP [15] and EGRET [18]) utilizing the structural information of proteins, whereas the remaining are sequence-based (PSIVER [24], ISIS [35], RF_PPI [7], SCRIBER [10], DELPHI [12]), relying solely on the sequence features of proteins.

**Fig. 3:**
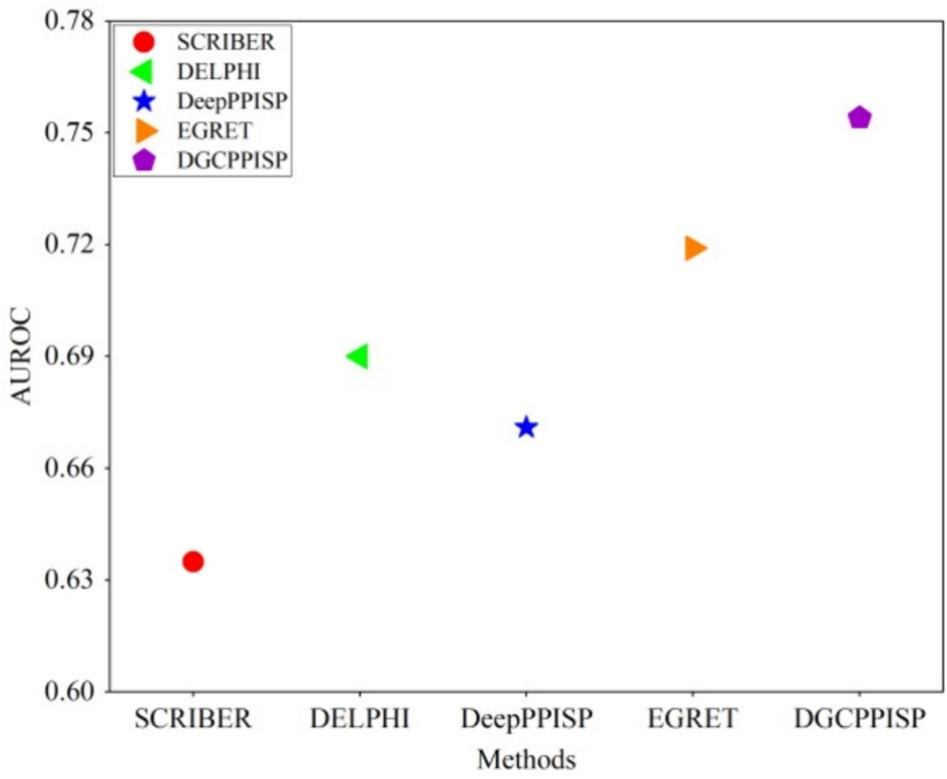
AUROC comparison of some methods on Dset_186_72_ PDB164

Table 1 showcases the results of DGCPPISP in comparison to other methods on Dset_186_72_PDB164. It is evident that our model outperforms in five of the six evaluated metrics. Even though our method exhibits a minor setback, relatively 2.4% lower than HN-PPISP in terms of recall, it still secures the second rank. Acknowledging the imbalanced nature of the PPI site prediction dataset, DGCPPISP demonstrates a conspicuous superiority in other performance metrics. Specifically, improvements in F1-measure, AUPRC and MCC are noted at 8.7%, 23.9% and 25.4% respectively. Comparisons with DELPHI, the current leading model among sequence-based methods, further underline DGCPPISP's dominance in all metrics, particularly AUPRC and MCC, exhibiting enhancements of 23.9% and 29.7% respectively. Interestingly, DGCPPISP surpasses structure-based methods even without relying on protein structure information. When compared to EGRET, a model employing GAT to incorporate protein structure, DGCPPISP outstrips it by 5.9%, 10.1% and 13.3% in F1-measure, AUPRC and MCC, respectively. These results imply that the superior performance of DGCPPISP may be attributed not only to the inherent advantages of dynamic graph convolution over GAT, but also to the integration of the ESM-2 embedding, assisting DGCPPISP in capturing covert information in protein feature representation. To further illustrate this, we select two representative methods from the sequence-based (SCRIBER and DELPHI) and structure-based (DeepPPISP and EGRET) categories and compare their AUROC values with the proposed DGCPPISP. As Fig. 3 indicates, DGCPPISP accomplishes superior prediction results on the AUROC performance index. In addition, we show the results of DGCPPISP on a version of the Dset_186_72_PDB164 with less than 20% sequence homology in Table 1, which persistently exhibit the commendable performance of our method.

**Table 1:** Comparison of PPI site prediction performance on Dset_186_72_PDB164

Method	ACC	Precision	Recall	F1	AUPRC	MCC
SPPIDER ^*^	0.622	0.209	0.459	0.287	0.230	0.089
ISIS	0.694	0.211	0.362	0.267	0.240	0.097
PSIVER	0.653	0.253	0.468	0.328	0.250	0.138
RF_PPI	0.598	0.173	0.512	0.258	0.210	0.118
SCRIBER	0.616	0.274	0.569	0.370	0.307	0.159
DeepPPISP ^*^	0.655	0.303	0.577	0.397	0.320	0.206
Attention-CNN ^*^	0.657	0.313	0.611	0.414	0.359	0.229
DELPHI	0.667	0.320	0.604	0.418	0.360	0.236
HN-PPISP ^*^	0.667	0.324	0.632	0.427	0.360	0.244
StackingPPINet	0.673	0.402	0.605	0.423	0.401	0.275
EGRET ^*^	0.715	0.358	0.561	0.438	0.405	0.270
DGCPPISP ^**^**^	0.684	0.349	0.611	0.459	0.424	0.294
DGCPPISP	0.718	0.372	0.617	0.464	0.446	0.306

“*” denotes methods using structural information. “^” means results on Dset_186_72_PDB164 with sequence homology less than 20%

**Fig. 4:**
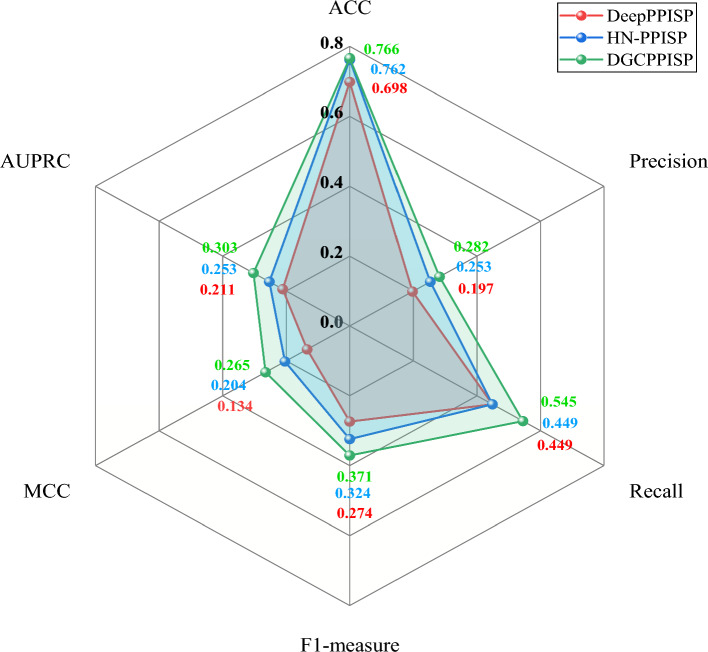
Prediction comparison on Dset_331

A more comprehensive evaluation of DGCPPISP is conducted by comparing it with two leading-edge methods, HN-PPISP and DeepPPISP, on Dset_331. As illustrated in Fig. 4, DGCPPISP surpasses both comparative methods across all six metrics, achieving full area coverage on the radar map. Specifically, in comparison to DeepPPISP, our model manifests improvements of 9.7% on ACC, 43.1% on Precision, 21.4% on Recall, 35.4% on F1-measure, 43.6% on AUPRC, and 97.8% on MCC, respectively. When juxtaposed with HN-PPISP, all six metrics of our model exhibit improvements by 0.5%, 11.5%, 21.4%, 14.5%, 19.8% and 29.9%, respectively. Moreover, the above two methods resort to PSSM for protein feature extraction, which is a time-consuming process. Our model circumvents this extraction operation through the transfer learning and solely utilizes conveniently extracted sequence encoding features while maintaining the prediction precision. This approach highlights the clear advantage of our method when the model's performance and the time cost of feature extraction are taken into account.

#### Comparison with traditional graph convolutional neural networks

**Fig. 5:**
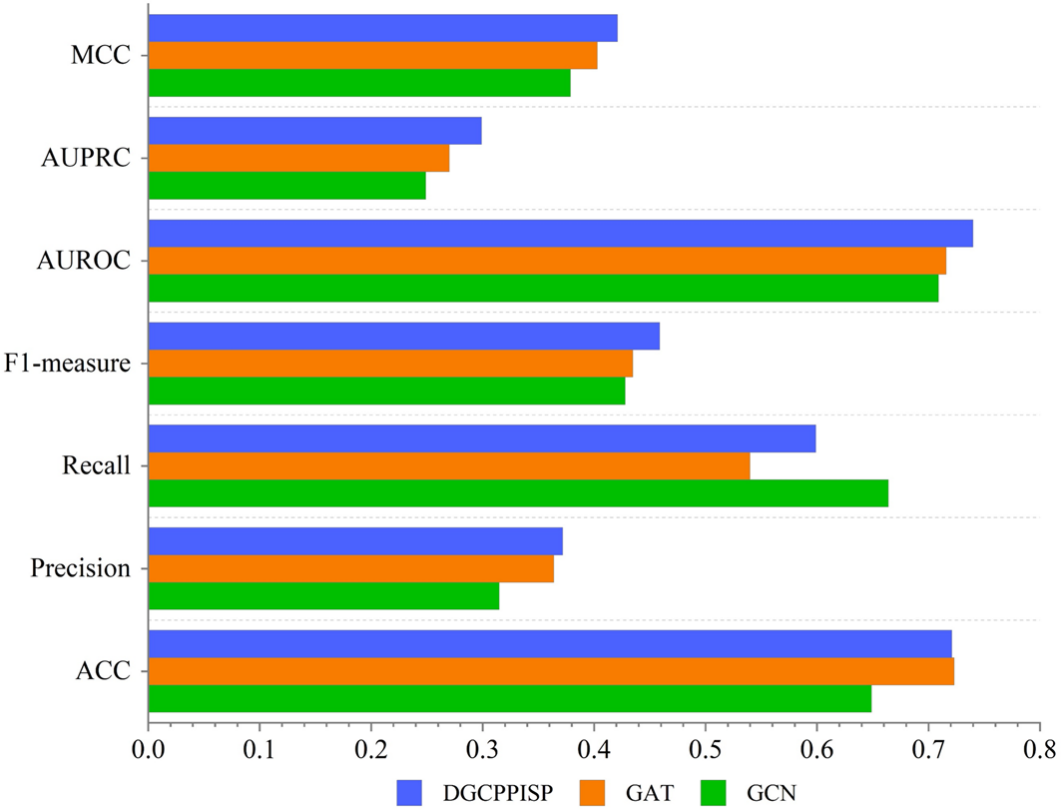
performance comparison with various graph neural networks

**Fig. 6:**
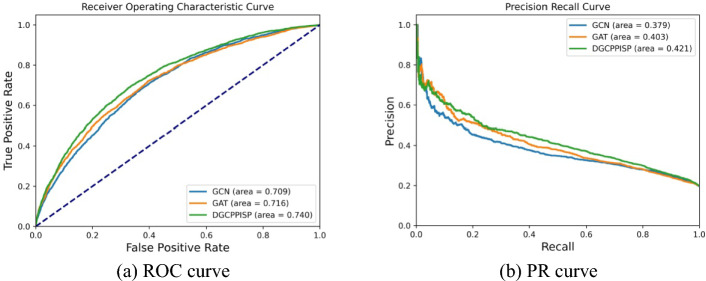
ROC and PR curves of three kinds of graph neural networks

In this subsection, we juxtapose the results of the dynamic graph convolutional neural network with the traditional graph neural network on PPI site prediction. We opt for two representative models for traditional graph neural networks: the graph convolutional network (GCN) [36] and the graph attention network (GAT) [19]. To establish a uniform comparison with the dynamic *k*-neighborhood graph of DGCPPISP, the node neighborhood range of both GCN and GAT is set to the same *k*, with nodes in the graph structure symbolizing the protein residues. Concurrently, we adhere to the adjacency construction method delineated in EGRET [18], electing the *k* residues with the nearest average atomic distance to the central node residues as their adjacent nodes. Concerning features, all five features in our model are selected as node features, meaning that all three graph networks have undergone the initial stage of information transfer. The performance of the three graph networks on Dset_186_72_ PDB164 is depicted in Fig. 5. Evidently, DGCPPISP outperforms the other two methods in five out of seven metrics, inclusive of four comprehensive performance metrics of primary concern. Although the GCN method does have an edge in Recall, its performance in the remaining six metrics is subpar in comparison. With the assistance of the multi-head attention mechanism, GAT exhibits superior overall performance compared to GCN, and its ACC is slightly higher than that of DGCPPISP. However, given the imbalance of the dataset, ACC alone is not the definitive metric for PPI site prediction. For the other metrics, GAT's results trail those of DGCPPISP. This experiment underscores the enhanced performance of the DGCPPISP model, based on a dynamic graph convolutional network, as opposed to traditional graph network methods GCN and GAT, which rely on fixed neighborhoods for PPI site prediction. We also graph the ROC and PR curves of the three graph convolutional neural networks on Dset_186_72_PDB164, as illustrated in Fig. 6. The discrepancies between these two curves also echo the superiority of DGCPPISP over the other two methods. We conclude it is attributed to dynamic graph convolutional neural networks’ ability to effectively adapt to non-stationary data by capturing time dependencies and dynamically update graph representations in the feature training process, thereby improving the prediction of PPI binding sites.

### Ablation study

#### Feature evaluation

**Fig. 7:**
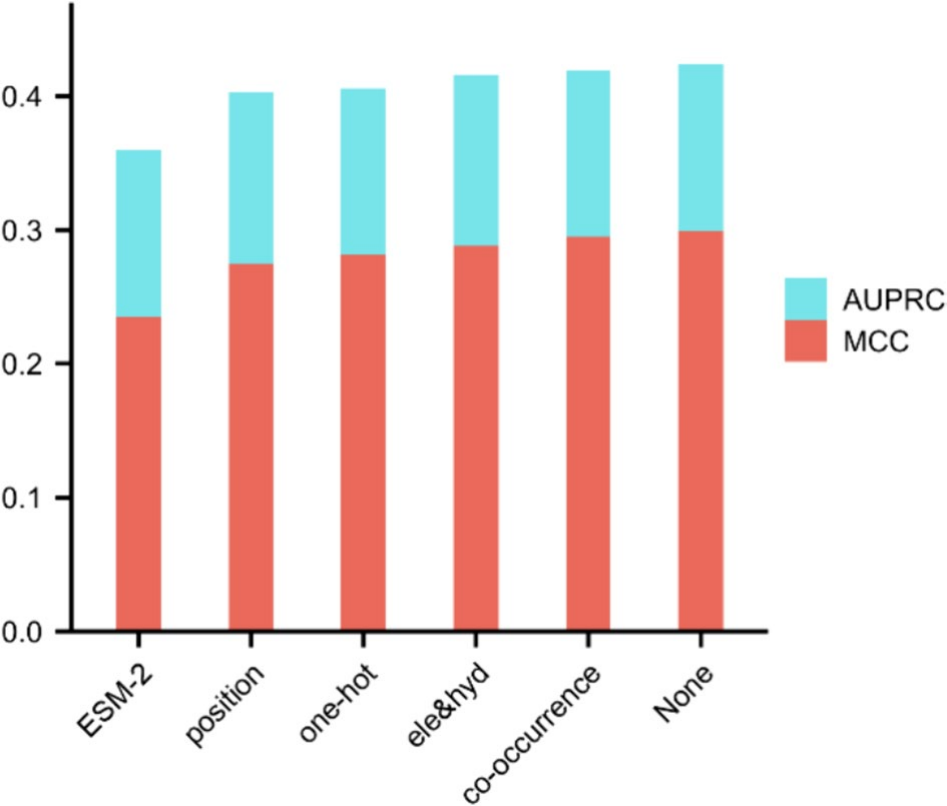
Effect of different features on model performance on Dset_186 _72_PDB164. Note that the x-axis shows the specific features that are removed. For amino acid co-occurrence similarity encoding and electrostaticity and hydrophobicity similarity encoding, they are named as co-occurrence and ele&hyd, respectively. None indicates that no features are removed

Our model harnesses a total of five protein sequence-based encodings as features. We gauge the impact of each feature on the performance of DGCPPISP through an ablation experiment. Specifically, we eliminate each feature in turn from the set of five features and subsequently test the model performance with a combination of the remaining four features. We then plot their AUPRC and MCC histograms on the primary dataset, Dset_186_72_PDB164 (notably, this experiment's results do not implicate the second stage of transfer learning, as no pre-training is performed on the protein-peptide binding residue dataset). As Fig. 7 illustrates, the model's performance on both metrics experiences a certain degree of decline after the removal of each feature. This validates that none of the features are superfluous, and all contribute substantially to the model's performance. Of particular note is ESM-2, which makes the most significant contribution to the model due to its abundant protein pre-training information. Without the aid of ESM-2 features, AUPRC reduces from 0.421 to 0.359, signifying a decline of 14.7%, and MCC descends from 0.299 to 0.230, indicating a 23.1% reduction. Remarkably, even in the absence of ESM-2, DGCPPISP still outperforms the majority of the other methods depicted in the ensuing Table 1.

#### Effectiveness analysis of transfer learning

To ascertain the performance of DGCPPISP at various stages of transfer learning, we perform an experiment on Dset_186_72_PDB164, as displayed in supplementary Table 2. It is observed that each stage of transfer learning contributes to varying degrees of performance enhancement in the overall model. For instance, the first stage of transfer learning yields the most significant improvements of 10.9%, 7.4%, 17.3%, and 28.3% in the final four metrics, respectively. This substantiates a high correlation with the rich features encompassed in ESM-2. The second stage of transfer learning contributes further improvements of 1.1%, 1.9%, 5.9%, and 2.3% respectively, which indicates that pre-trained model parameters offer beneficial prediction performance compared to random parameter selection in feature extraction and representation. We also depict the ROC and PR curves corresponding to the three transfer learning stages to intuitively exhibit the impact of transfer learning on DGCPPISP, as illustrated in supplementary Fig. 8. It is discernible that the ROC and PR curves drawn using the model with the complete two-stage transfer learning are generally positioned above the curves of models with the other two stages separately, signifying excellent performance.

**Table 2:** Performance comparison of different stages of transfer learning on Dset_186_72_PDB164

Method	ACC	Precision	Recall	F1	AUROC	AUPRC	MCC
Model_RAW	0.673	0.321	0.586	0.414	0.689	0.359	0.233
Model_Stage1	0.721	0.372	0.599	0.459	0.740	0.421	0.299
Model_Stage2	0.718	0.372	0.617	0.464	0.754	0.446	0.306

Model_RAW denotes that no transfer learning is used; Model_Stage1 denotes the first stage of transfer learning; Model_Stage2 denotes the full method using two-stage transfer learning

**Fig. 8:**
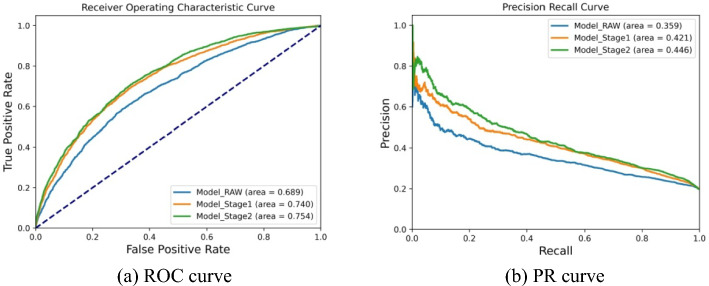
Comparison of three states of transfer learning

The same experiment is subsequently conducted on Dset_331, and the results are presented in supplementary Table 3. After employing the first stage of transfer learning, the F1-measure, AUROC, AUPRC, and MCC of DGCPPISP are elevated by 8.4%, 6.3%, 19.1%, and 17.8% respectively. Further introduction of the second stage of transfer learning increases the performance of DGCPPISP on the four indicators by 6.0%, 0.8%, 1.3%, and 11.3% respectively, thereby demonstrating the effectiveness of transfer learning in our model.

**Table 3:** Performance comparison of different stages of transfer learning on Dset_331

Method	ACC	Precision	Recall	F1	AUROC	AUPRC	MCC
Model_RAW	0.744	0.243	0.480	0.323	0.693	0.251	0.202
Model_Stage1	0.778	0.279	0.471	0.350	0.737	0.299	0.238
Model_Stage2	0.766	0.282	0.545	0.371	0.743	0.303	0.265

#### The effect of different k-neighborhood

In our model, dynamic graph convolution is employed to form a "dynamic graph" by constructing the neighborhood of the central node via a *k*-nearest neighbors algorithm based on the "feature distance" prior to the EdgeConv operation. Consequently, the size of the *k* setting determines the field of view of the EdgeConv operation and to a certain extent influences the model performance. To scrutinize the impact of neighborhood range on the model's performance, we assign six diverse ranges of neighborhoods from small to large and document the performance of DGCPPISP on each metric. The minimum value of *k* is fixed at 1, implying that each central node has only a single neighborhood node. We illustrate the variance of four crucial metrics as a line graph in Additional file 1: Fig. S1. It is apparent that all four metrics achieve their optimal results at *k* = 10, and although the AUPRC exhibits a marginal increase at* k* = 40, overall, the values of the metrics trend downward as the neighborhood range contracts and expands. We deduce that when the *k*-neighborhood range is too minimal, the EdgeConv's field of view is constrained, preventing the full exploration of relationships between the central node and other nodes (such as those long "feature distance" dependencies implied in the high-dimensional semantic space), which influences the effective feature extraction of DGCPPISP. When the *k*-neighborhood is excessively large, the neighborhoods of each node in the deep network may closely approximate each other, leading to the updated nodes exhibiting similarity in features and subsequently affecting the robustness of the model.

#### Effect of different kernel size for Conv_encoder

Within the DGCPPISP model, we have integrated a Conv_encoder module, constructed via one-dimensional convolution, aimed at both elevating and reducing the dimensionality of features. To pinpoint an optimal convolution kernel size, we execute a comparative experiment encompassing disparate convolution kernel sizes, as presented in Additional file 1: Table S4. It is observed from the table that when the convolution kernel size of Conv_encoder is 3, it takes the lead in five metrics, thus being incorporated as the parameter setting in our model.

#### Impact of different protein length

**Fig. 9:**
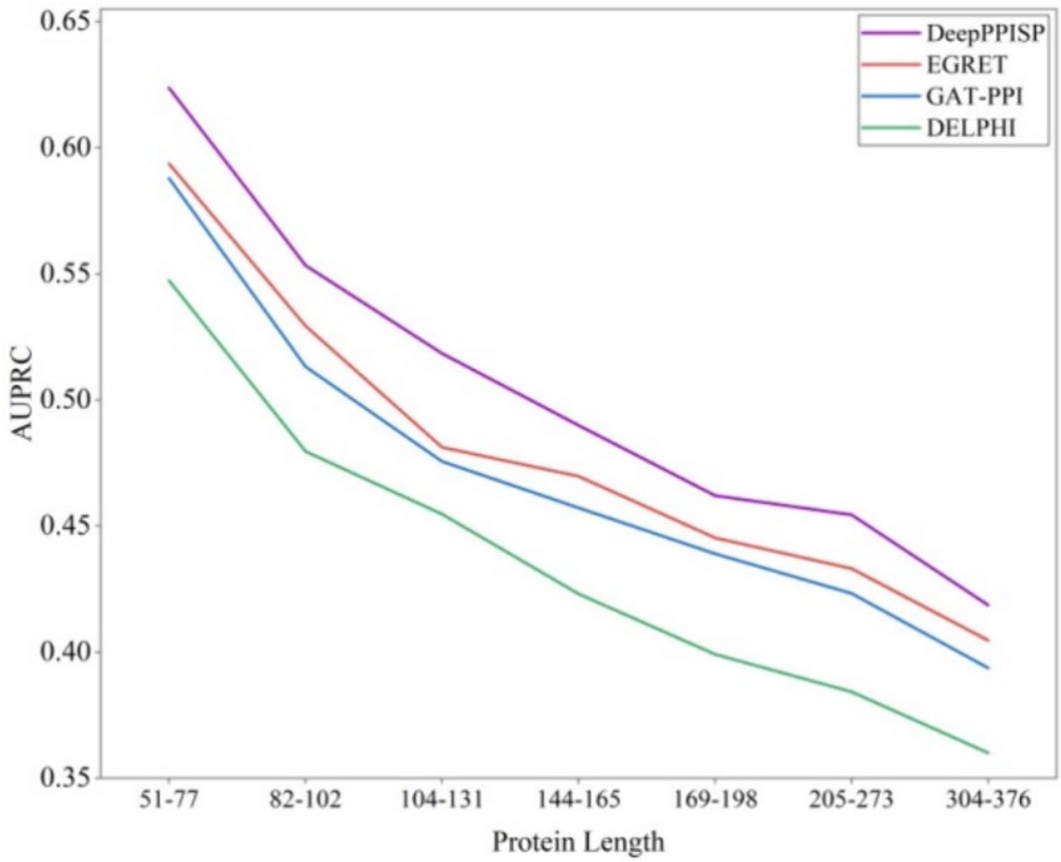
AUPRC on various lengths of proteins (The x-axis represents different protein subsets according to length)

A hyperparameter of our model, the dynamic neighborhood range* k*, is typically determined by the length of the protein. Hence, in this section, we maintain the *k* value at 10 (the superior parameter selected in Sect. 3.3.3) to scrutinize the influence of protein length on DGCPPISP. Utilizing Dset_186_72_PDB164 as a representative example, we partition the 70 proteins used for testing into seven non-overlapping intervals based on sequence length, ensuring each interval encompasses 10 proteins. The AUPRC of DGCPPISP is calculated across the seven intervals and subsequently compared with popular methods. These results are illustrated in Fig. 9. For the methods compared, GAT-PPI is a derivative of EGRET and essentially an EGRET version devoid of the aggregated edge feature. It is discernible that, although for DGCPPISP and other three methods, their AUPRC scores consistently decline as protein sequence length increases, the overall performance of DGCPPISP across the seven intervals outstrips other methods.

### Visualization analysis

**Fig. 10:**
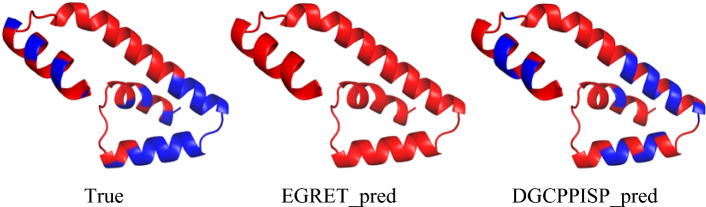
Visualization comparison of DGCPPISP and EGRET on PDB ID: 3VDO, Chain B. Note that the red area indicates the PPI site, and the blue area indicates the non-PPI site

To further appraise the capacity of DGCPPISP in PPI site prediction, we additionally furnish a visualization comparison of DGCPPISP and EGRET. Selecting four proteins (PDB ID: 1MAF, Chain F; PDB ID: 3D7V, Chain A; PDB ID: 3VDO, Chain B; PDB ID: 3W2W, Chain B, respectively) from Dset_186_72_PDB164's test set for our experiment, we visualize the true PPI site (True), the PPI site predicted by EGRET (EGRET_pred), and the PPI site predicted by DGCPPISP (DGCPPISP_pred), as depicted in Additional file 1: Fig. S2. It is evident that the visualization results generated by DGCPPISP markedly surpass EGRET and align more closely with the true cases, particularly in Fig. 10, where EGRET identifies all residues of the 3VDO protein as PPI sites, starkly contradicting the true label, whereas DGCPPISP demonstrates superior precision on this protein.

**Fig. 11:**
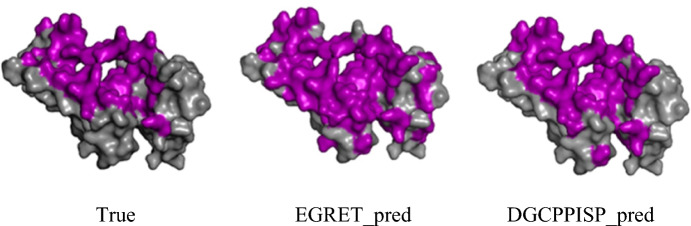
Surface visualization comparison of DGCPPISP and EGRET on PDB ID: 3L9F, Chain A, where purple indicates PPI sites and gray indicates non-PPI sites

Furthermore, we supply visual prediction results in the form of protein surfaces, selecting three proteins from Dset_331 for comparison with the true PPI site, the results of DGCPPISP, and EGRET. These results, displayed in Fig. 11 and Additional file 1: Fig. S3, further substantiate that our model's results more closely resemble the true PPI site result.

## Conclusion

This paper introduces DGCPPISP, an innovative sequence-based PPI site prediction method. It implements a two-stage transfer learning strategy to enhance the overall performance of DGCPPISP by equipping the model with prior knowledge from both the feature input and initial parameters, respectively. Within the network framework, DGCPPISP forms a dynamic graph convolution module to supplant a traditional graph convolutional neural network, thereby augmenting the model's capability to extract node features through evolving dynamic neighborhoods. Comprehensive experimental analysis and comparison corroborate the efficacy of the proposed model and its superior performance over other popular methods.

Certainly, there remains room for enhancement. Currently, our model is predominantly reliant on protein sequence information. Future work will explore the integration of sequence and structure information for PPI site prediction. Additionally, our model's main contribution comes from transfer learning, where the protein-peptide binding site prediction is luckily found to be beneficial for PPI site prediction. Identifying other related prediction tasks more suited for PPI site prediction will form another aspect of our future work. Lastly, the PPI site prediction model is an unbalanced dataset with more negative than positive samples. Expanding the number of positive samples and constructing a higher quality dataset will remain a key focus in future endeavors.

### Supplementary Information

**Additional file 1: Table S1**. Summary of datasets. **Table S2**. Statistics of lengths of all sequences in the experiment. **Table S3**. Classification of amino acids based on electrostatic and hydrophobicity. **Table S4**. Performance comparison with different sizes of convolution kernels. **Fig. S1**. Impact of different neighborhood ranges on model performance. **Fig. S2**. Visualization comparison of DGCPPISP and EGRET on proteins from Dset_186_72_PDB164. **Fig. S3**. Three examples of PPI site prediction on dataset Dset_331.

## Data Availability

The source code and data information is publicly available at https://github.com/Mrfengdashen/DGCPPISP.

## Abbreviations

PPI: Protein–protein interaction
RF: Random forest
LR: Logistic Regression
GAT: Graph attention network
PSSM: Position-specific scoring matrix
MLP: Multilayer perceptron
AUROC: Area under the receiver operating characteristic curve
AUPRC: Area under the precision-recall curve
MCC: Matthews correlation coefficient
GCN: Graph convolutional network
