# Supplementary material for: DGCPPISP: a PPI site prediction model based on dynamic graph convolutional network and two-stage transfer learning
Source: BMC Bioinformatics. 2024 Jul 31;25:252. doi: 10.1186/s12859-024-05864-w (PMC11293074; doi:10.1186/s12859-024-05864-w)
Supplement: Supplementary file 1 — Additional file 1: Table S1. Summary of datasets. Table S2. Statistics of lengths of all sequences in the experiment. Table S3. Classification of amino acids based on electrostatic and hydrophobicity. Table S4. Performance comparison with different sizes of convolution kernels. Fig. S1. Impact of different neighborhood ranges on model performance. Fig. S2. Visualization comparison of DGCPPISP and EGRET on proteins from Dset_186_72_PDB164. Fig. S3. Three examples of PPI site prediction on dataset Dset_331. [file 12859_2024_5864_MOESM1_ESM.docx]

**DGCPPISP：A PPI site prediction model based on dynamic graph convolutional network and two-stage transfer learning**

**(Supplementary materials)**

#### Supplementary Note

| **Algorithm** DGCPPISP optimization |
| --- |
| **Input** the two labeled data sets $T_{d}$ (pre-train) and $T_{s}$, and the number of iterations $N_{d}$ and $N_{s}$ **Initialize** the initial weight vector $\theta$, feature of residue $r$  **Training**  For *t* = 1, …, $N_{d}$  1: Using initial features and constructing dynamic graph structures through KNN  2: message passing on dynamic graphs and update node features $r_{i}{\to r}_{i}^{'}$  3: Calculate the loss on $T_{d}$  4: Update the weight vector$\theta$  For *t* = 1, …, $N_{s}$  1: Using initial features and constructing dynamic graph structures through KNN  2: message passing on dynamic graphs and update node features $r_{i}{\to r}_{i}^{''}$  3: Calculate the loss on $T_{s}$  4: Update weight vector $\theta$  **Predicting PPI binding sites** |

#### Supplementary Tables

**Table S1**. Summary of datasets.

| Datasets | Dataset_trans | | | Dset_186_72_PDB164 | | Dset_331 | |
| --- | --- | --- | --- | --- | --- | --- | --- |
|  | training | testing | | training | testing | training | testing |
| Proteins | 1154 | | 125 | 352 | 70 | 254 | 77 |
| Residues total | 276822 | | 30870 | 73181 | 11791 | 65043 | 18632 |
| Interaction sites | 15030 | | 1719 | 11079 | 2332 | 8890 | 2365 |
| Non-interaction sites | 261792 | | 29151 | 62102 | 9459 | 56153 | 16267 |
| Binding % of total | 5.43 | | 5.57 | 15.14 | 19.78 | 13.67 | 12.29 |

**Table S2**. Length statistics of all sequences in the experiment.

| Sequence  length: | 1-  100 | 100-  200 | 200-  300 | 300-  400 | 400-  500 | 500-  600 | 600-  700 | 700  + |
| --- | --- | --- | --- | --- | --- | --- | --- | --- |
| Dataset 1 | 85 | 176 | 68 | 56 | 23 | 7 | 4 | 3 |
| Dataset 2 | 25 | 99 | 100 | 68 | 28 | 6 | 5 | 0 |

**Table S3**. Classification of amino acids based on electrostatic and hydrophobicity.

| No. | Volume Scale | Dipole Scale | Class |
| --- | --- | --- | --- |
| 1 | **-** | **-** | A, G, V |
| 2 | **+** | **-** | I, L, F, P |
| 3 | **+** | **+** | Y, M, T, S |
| 4 | **+** | **++** | H, N, Q, W |
| 5 | **+** | **+++** | R, K |
| 6 | **+** | **+’+’+’** | D, E |
| 7 | **+** | **+’’** | C |

Note：Dipole Scale: -, Dipole<1.0; +, 1.0< Dipole<2.0; ++, 2.0<Dipole<3.0; +++, Dipole>3.0; +'+'+', Dipole>3.0 in the opposite direction; +'', cysteine can form disulfide bonds, so it is classified into a separate category. Volume Scale: -, Volume<50 Å^3^; +, Volume>50 Å^3^.

**Table S4**. Performance comparison with different sizes of convolution kernels.

| Kernel size | ACC | Precision | Recall | F1 | AUROC | AUPRC | MCC |
| --- | --- | --- | --- | --- | --- | --- | --- |
| 1 | 0.702 | 0.353 | 0.614 | 0.449 | 0.734 | **0.424** | 0.283 |
| 3 | **0.721** | **0.372** | 0.599 | **0.459** | **0.740** | 0.421 | **0.299** |
| 5 | 0.697 | 0.347 | 0.605 | 0.442 | 0.723 | 0.389 | 0.272 |
| 7 | 0.678 | 0.351 | 0.625 | 0.450 | 0.732 | 0.406 | 0.284 |
| 9 | 0.696 | 0.352 | **0.640** | 0.454 | 0.729 | 0.389 | 0.290 |

#### Supplementary Figures


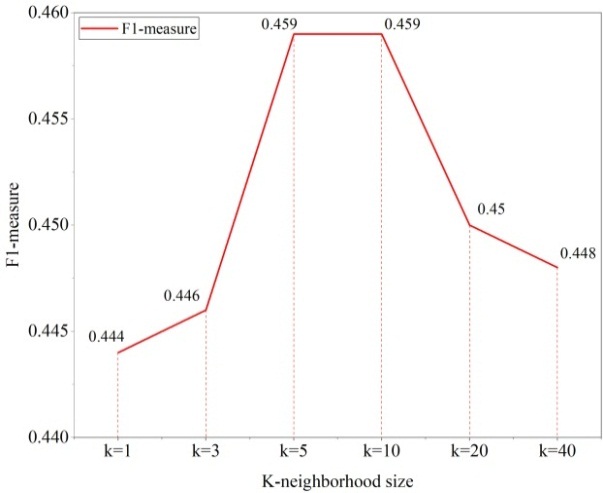

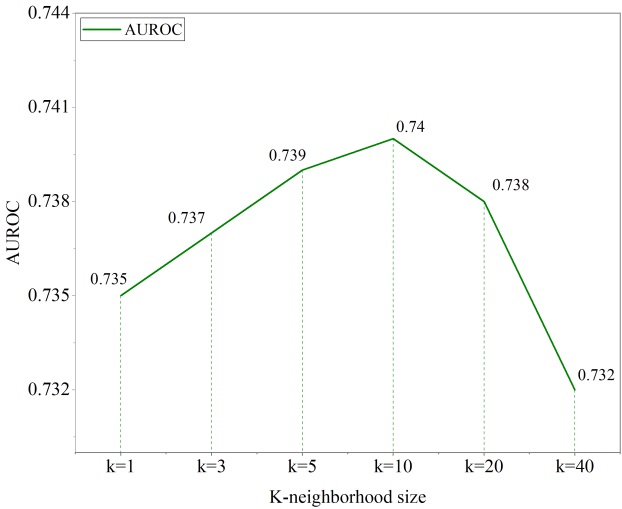


(a) F1-measure (b) AUROC


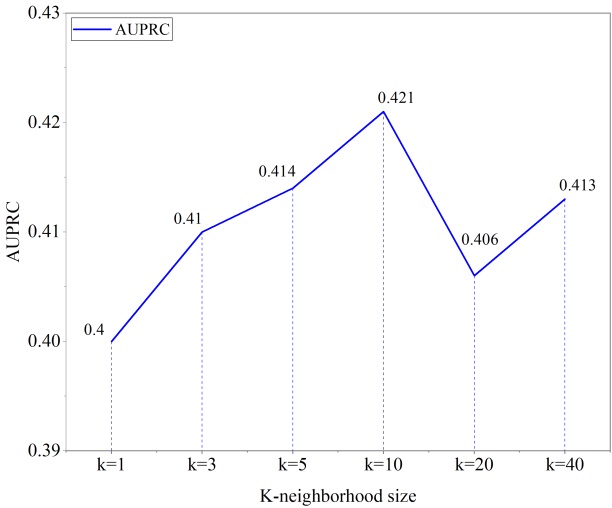

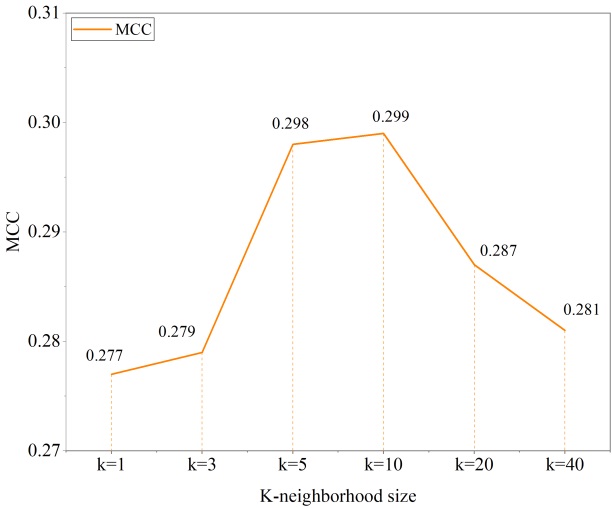


(c) AUPRC (d) MCC

**Fig. S1**. Impact of different neighborhood ranges on model performance.


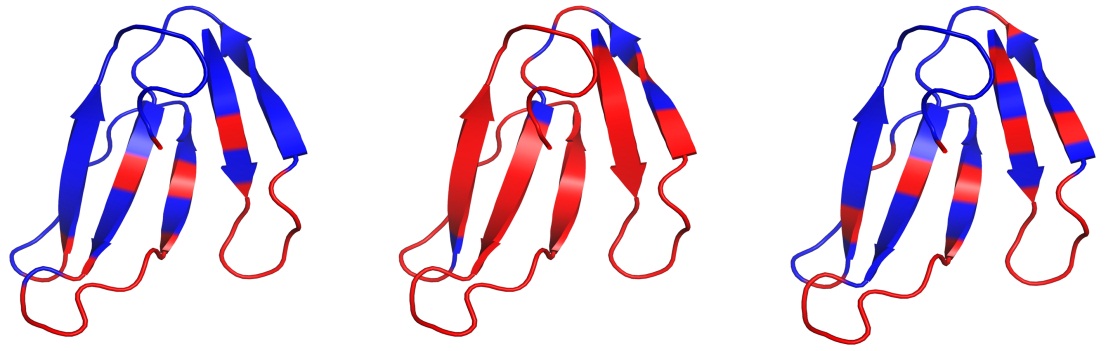


True EGRET_pred DGCPPISP_pred

(a) PDB ID: 1MAF, Chain F


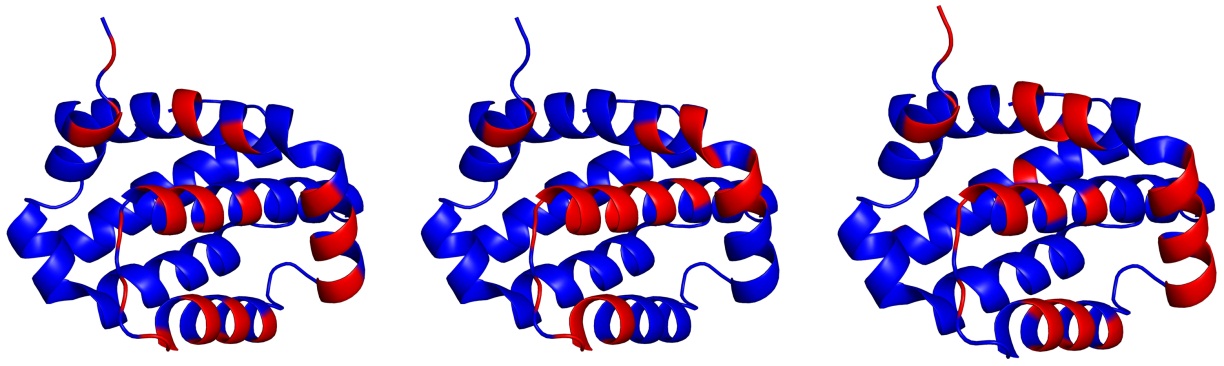


True EGRET_pred DGCPPISP_pred

(b) PDB ID: 3D7V, Chain A


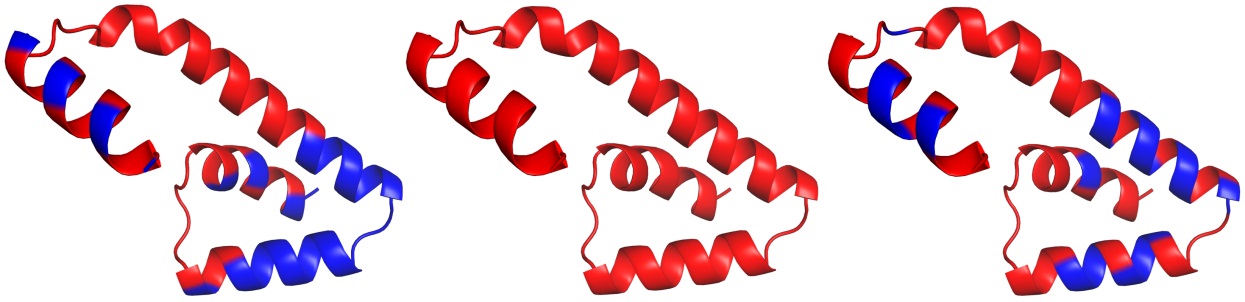


True EGRET_pred DGCPPISP_pred

(c) PDB ID: 3VDO, Chain B


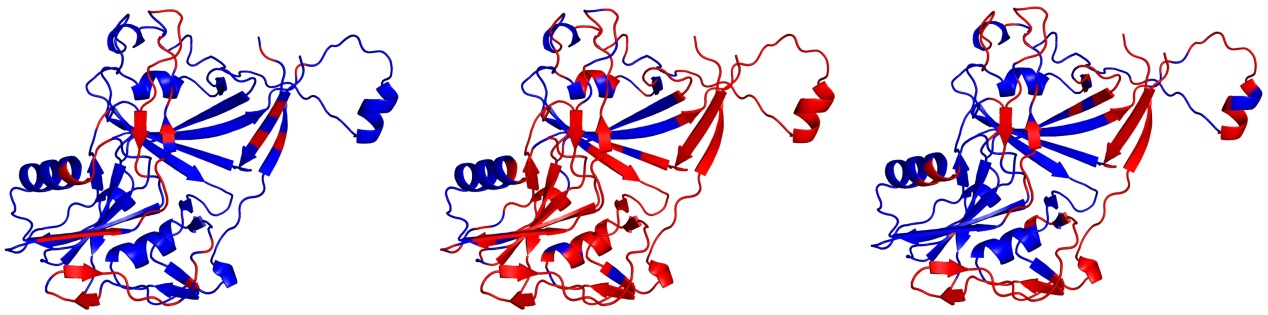


True EGRET_pred DGCPPISP_pred

（d）PDB ID: 3W2W, Chain B

**Fig. S2**. Visualization comparison of DGCPPISP and EGRET on proteins from Dset_186_72_PDB164. Note that the red area indicates the PPI site, and the blue area indicates the non-PPI site.


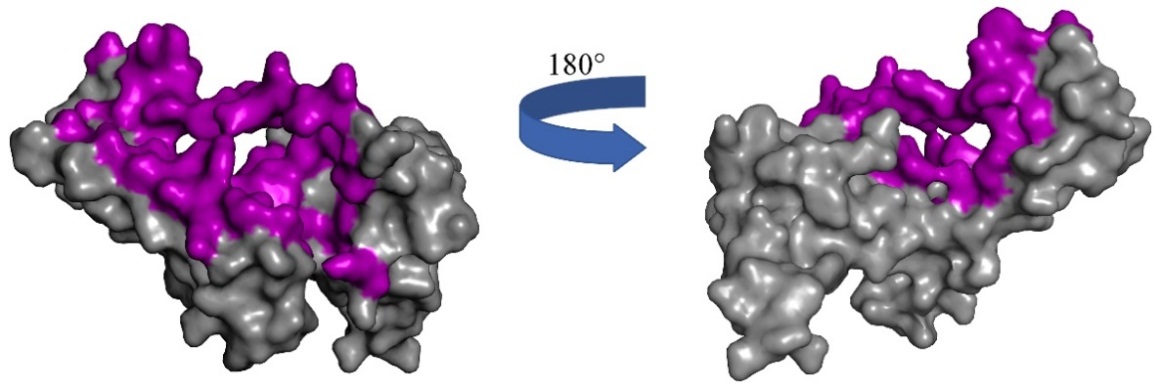


(i) (ii)

True labels.


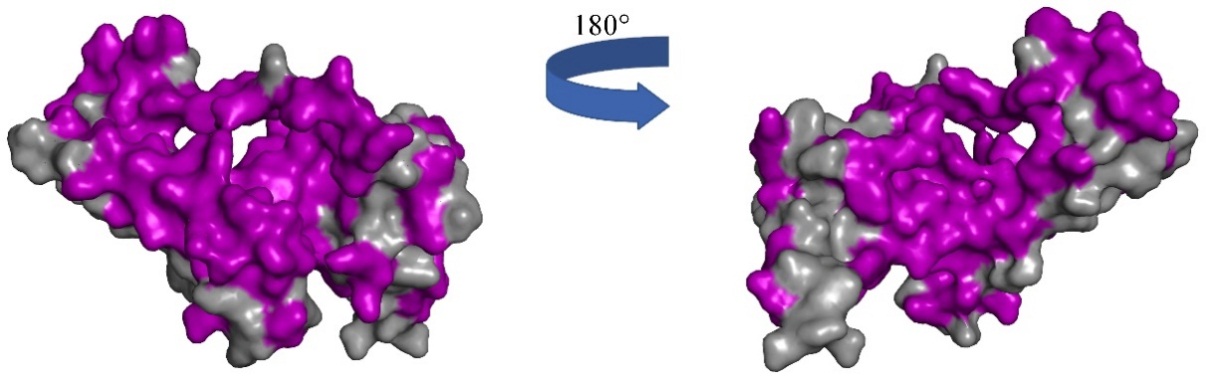


(i) (ii)

EGRET_pred result.


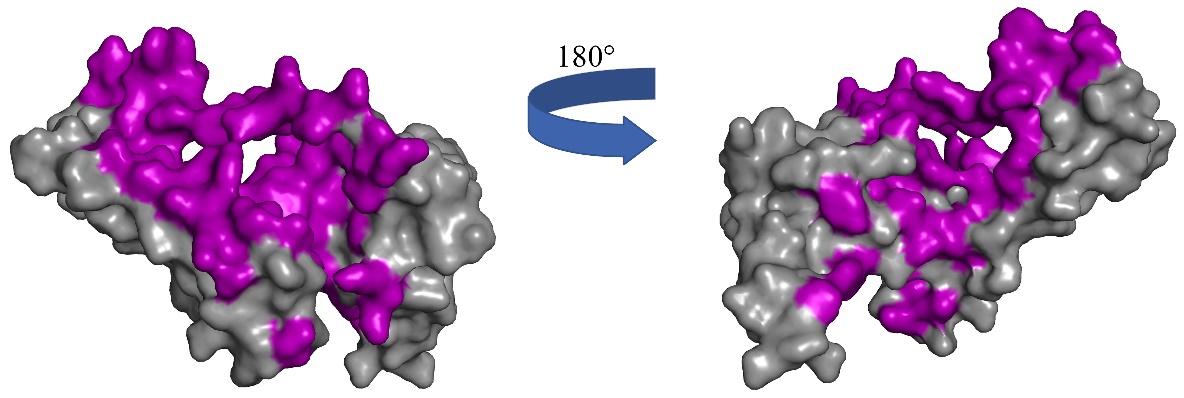


(i) (ii)

DGCPPISP_pred result.

(a) PDB ID: 3L9F, Chain A.


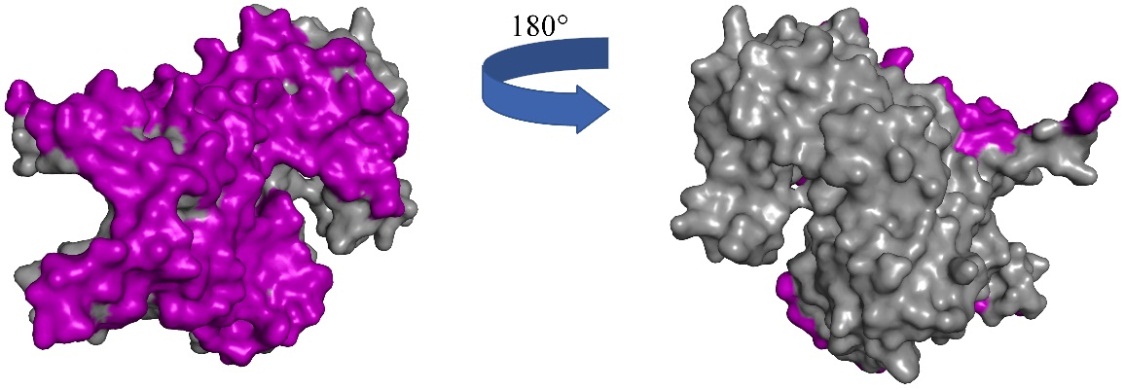


(i) (ii)

True labels.


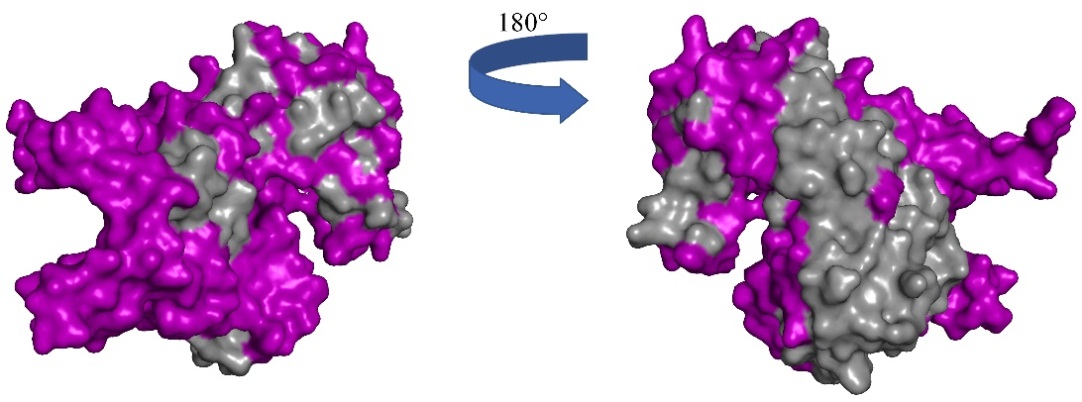


(i) (ii)

EGRET_pred result.


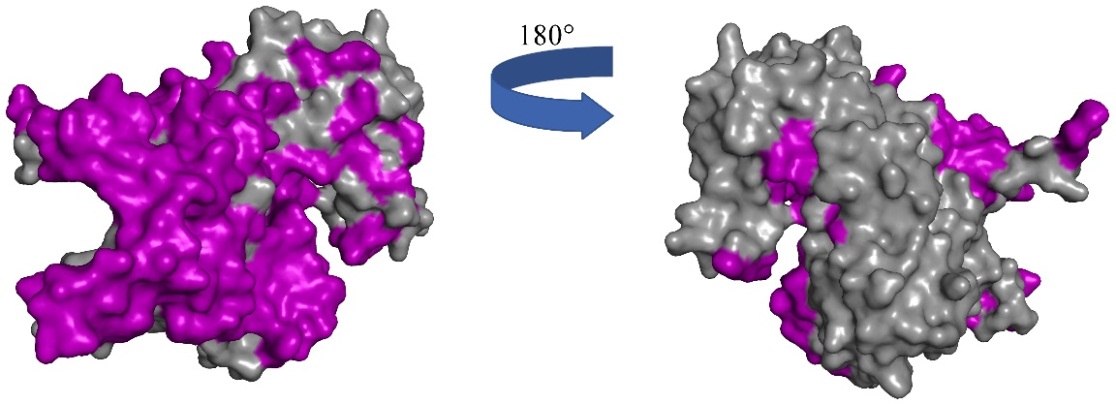


(i) (ii)

DGCPPISP_pred result.

(b) PDB ID: 5X2W, Chain D.


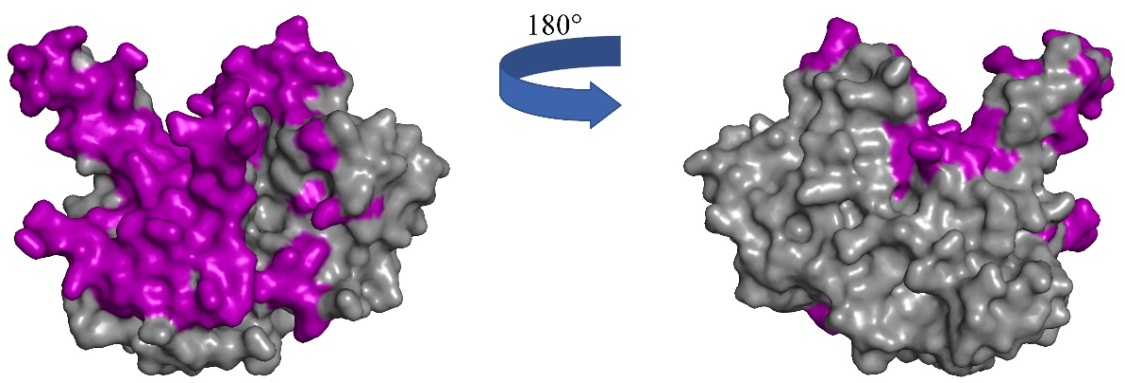


(i) (ii)

True labels.


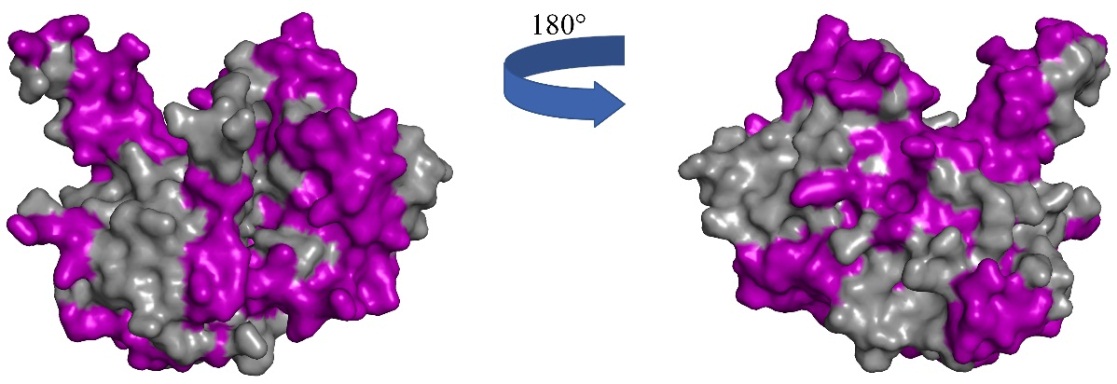


(i) (ii)

EGRET_pred result.


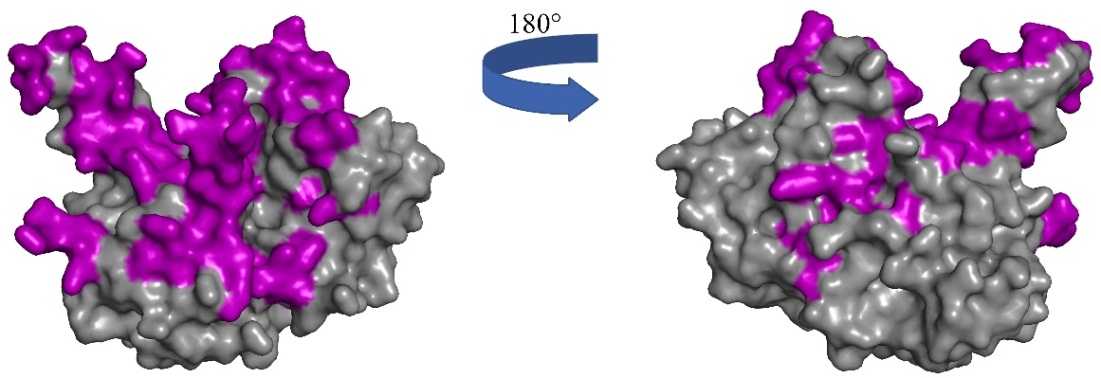


(i) (ii)

DGCPPISP_pred result.

(c) PDB ID: 2PHN, Chain B.

**Fig. S3**. Three examples of PPI site prediction on dataset Dset_331. The picture (ii) is obtained by rotating 180 degrees from picture (i), where purple indicates PPI sites and gray indicates non-PPI sites.
